# A Deep Learning-Based Intrusion Detection System for MQTT Enabled IoT

**DOI:** 10.3390/s21217016

**Published:** 2021-10-22

**Authors:** Muhammad Almas Khan, Muazzam A. Khan, Sana Ullah Jan, Jawad Ahmad, Sajjad Shaukat Jamal, Awais Aziz Shah, Nikolaos Pitropakis, William J. Buchanan

**Affiliations:** 1 Department of Computer Sciences, Quaid-i-Azam University, Islamabad 44000, Pakistan; mkhan@cs.qau.edu.pk; 2 School of Computing, Edinburgh Napier University, Edinburgh EH10 5DT, UK; S.Jan@napier.ac.uk (S.U.J.); N.Pitropakis@napier.ac.uk (N.P.); B.Buchanan@napier.ac.uk (W.J.B.); 3 Department of Mathematics, College of Science, King Khalid University, Abha 61413, Saudi Arabia; shussain@kku.edu.sa; 4 Department of Electrical and Informational Engineering (DEI), Polytechnic University of Bari, 70125 Bari, Italy; awais.shah@poliba.it

**Keywords:** MQTT, IDS, IoT, security, classification

## Abstract

A large number of smart devices in Internet of Things (IoT) environments communicate via different messaging protocols. Message Queuing Telemetry Transport (MQTT) is a widely used publish–subscribe-based protocol for the communication of sensor or event data. The publish–subscribe strategy makes it more attractive for intruders and thus increases the number of possible attacks over MQTT. In this paper, we proposed a Deep Neural Network (DNN) for intrusion detection in the MQTT-based protocol and also compared its performance with other traditional machine learning (ML) algorithms, such as a Naive Bayes (NB), Random Forest (RF), k-Nearest Neighbour (kNN), Decision Tree (DT), Long Short-Term Memory (LSTM), and Gated Recurrent Units (GRUs). The performance is proved using two different publicly available datasets, including (1) MQTT-IoT-IDS2020 and (2) a dataset with three different types of attacks, such as Man in the Middle (MitM), Intrusion in the network, and Denial of Services (DoS). The MQTT-IoT-IDS2020 contains three abstract-level features, including Uni-Flow, Bi-Flow, and Packet-Flow. The results for the first dataset and binary classification show that the DNN-based model achieved 99.92%, 99.75%, and 94.94% accuracies for Uni-flow, Bi-flow, and Packet-flow, respectively. However, in the case of multi-label classification, these accuracies reduced to 97.08%, 98.12%, and 90.79%, respectively. On the other hand, the proposed DNN model attains the highest accuracy of 97.13% against LSTM and GRUs for the second dataset.

## 1. Introduction

Internet-of-Things (IoT) augments the physical objects (usually referred to as IoT nodes) with internet connectivity such that they can collect and share data with other nodes in the network without human interventions. To enable the secure and reliable exchange of data among IoT nodes, different communication and messaging protocols have been developed, such as Constrained Application Protocol (CoAP), Advanced Message Queuing Protocol (AMQP), Message Queuing Telemetry Transport (MQTT), and Extensible Messaging Presence Protocol (XMPP) [1]. Among all, MQTT has been widely used in smart homes [2,3,4], agricultural IoT [5,6], and industrial applications [7], etc. The reasons include support for communication on low bandwidths, low memory requirements, and reduced packet loss [1,8,9].

**Figure 1:**
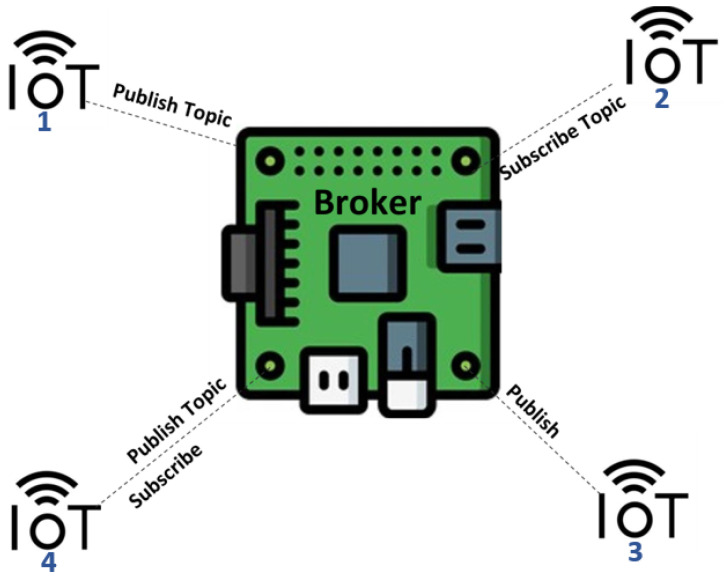
IoT communication via MQTT protocol.

MQTT communication protocol consists of four major components, including broker (central device), clients (IoT nodes), topic, and message. The topic in the MQTT protocol also contains information about the source and destination nodes of transmission messages among the networks. These topics are structured using the forward slash delimiter (/) with messages consisting of data gathered by IoT sensors. Using the MQTT messaging transport protocol, every node has three main associated tasks: topic selection, topic publication, and topic subscription [9,10]. Primarily, the IoT nodes (clients) of MQTT communicate with each other via a central node called a broker; a broker can be working on the edge, i.e., a local broker, or it can be on the cloud, i.e., a remote broker. It allows IoT nodes to publish or subscribe topics or publish and subscribe at the same time if the node functionality allows, as shown in Figure 1 in general. For example, a Passive Infrared Sensor (PIS) for motion detection publishes sensed data to the broker that is subscribed by a camera. As soon as the PIS detects motion, the information is sent to the camera for further action.

The IoT has been identified as the most vulnerable network to be attacked by external, as well as internal, attackers [11,12,13]. External attackers try to corrupt the system from outside the networks. On the contrary, internal attackers operate from inside the network under threat. The internal attackers, however, can access information easily as compared to external attackers. In either case, prior to initiating an attack, the attackers usually gather information to check the vulnerability of the network or system using different tools, such as Masscan, Network Mapper (NMAP), or Shodan [14]. For example, the paper [15] presents a case scenario of an attacker using penetrating testing tools to collect information of brokers through the Shodan tool. Furthermore, the Shodan tool provided connection codes that indicate whether a broker needs authentication or not.

**Figure 2:**
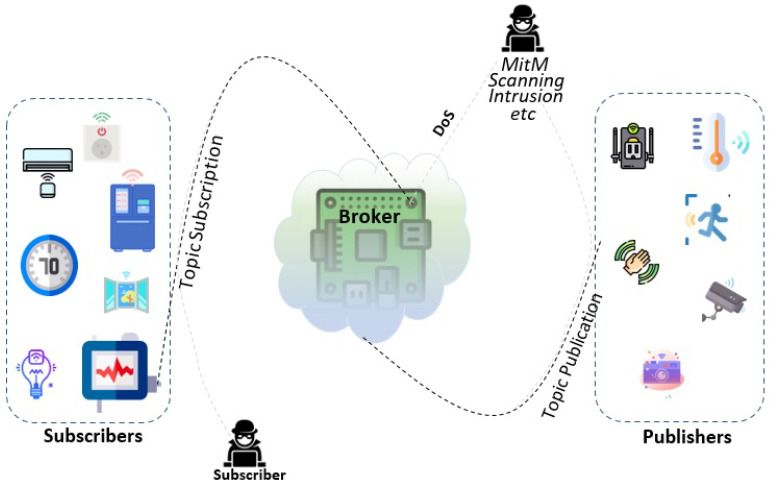
Attack scenario over MQTT protocol.

The different types of threats to the broker in an MQTT protocol are illustrated in Figure 2. As an example, by breaching the broker security and making to all topics, an attacker can expose critical information of the system. Similarly, if an attacker publishes a topic the same as any other publisher, it can control the subscribers of a given topic [15]. For example, streetlights can be subscribers to a valid publisher in a smart streetlight system [16]. An attacker connected to its broker can generate and send the wrong information over to control these streetlights. In addition, an internal attacker can compromise the integrity of MQTT data packets as they can have an opportunity of analysing and modifying them.

Studies show that attackers usually target central communication devices, i.e., brokers, in MQTT-based IoT systems. Denial-of-Service (DoS) [17], Man-in-the-Middle (MitM), scanning, and Intrusion are a few examples of common attacks on brokers [15,17,18,19]. In principle, the MQTT client starts a connection with a broker by sending a connect packet, and, since MQTT works on top of TCP/IP, the broker sends connection acknowledgement (connack). After receiving acknowledgement, the client starts data transmission to the broker. MQTT protocol can provide three levels of Quality of Service (QoS) that define the level of agreement and the assurance of successful communication between a transmitter and receiver in the network. The QoS level 0 has no acknowledgement mechanism in communication between the sender and receiver. [20]. In addition, an internal attacker sends multiple messages with QoS1 and QoS2 to make the broker busy in acknowledgements, thereby imposing a DoS attack [18].

Machine learning (ML) has shown efficiency in different application areas, including intrusion detection systems for IoT [21,22,23]. Some researchers opine that ML has the potential to not only efficiently detect but also predict the attacks given efficient data have been used to train them. Therefore, in this paper, we propose an Intrusion Detection System (IDS) for MQTT protocol based on the ML algorithm, i.e., a Deep Neural Network (DNN). The proposed DNN algorithm is evaluated on the latest dataset named MQTT-IoT-IDS2020 and the dataset (https://joseaveleira.es/dataset; access date was 8 July 2021) discussed in [24] that contains three well-known attacks: MitM, Intrusion, and DoS over MQTT. The selected datasets [24,25] are generated in an MQTT simulated environment. In MQTT-IoT-IDS2020, there are three abstract-level features, such as Packet-flow, Uni-flow, and Bi-flow, as mentioned in [25]. Detailed statistics of this dataset will be found in the upcoming section of this paper. The contributions can be summarised as follows.

- A DNN is proposed in this work for intrusion detection in MQTT-based protocol. Additionally, a number of ML models have been evaluated and compared for three different scenarios, including Bi-flow, Uni-flow, and Packet-flow, of abstract levels in the MQTT-IoT-IDS2020 dataset. The evaluation has been performed for binary as well as multi-class classification.
- The performance of the proposed DNN model is also evaluated for different attacks, including DoS, Intrusion, and MitM, in another dataset [24].

The remainder of this paper is organised as follows. Section 2 presents a literature review and a detailed discussion about related works. Section 3 provides a detailed explanation of the proposed intrusion detection system and other classical ML models. Section 4 illustrates the experimental setup, dataset selection criteria, results, and a discussion of the results. Section 5 concludes the paper and highlights potential future directions.

## 2. Related Works

IoT security is an open research area currently being addressed by researchers around the globe. Different security-enhancing methods have been proposed to protect IoT against anomalous adversarial attacks. These methods commonly aim at detecting intruders in the network by monitoring network activities, such as data flow rate. Here, a short literature review is presented to put forward current advances in IoT security, with a focus on intrusion detection systems targeting the MQTT messaging protocol of IoT. The authors of [26] presented an attack detection strategy for MQTT protocols based on a process tree. It models the network behaviour in terms of hierarchical branches of a tree, where it is further applied to detect attacks or anomalous behaviours. The model is evaluated using a detection rate where a total of four common types of attacks are induced in the network. However, newly developed adversarial attacks and intrusions have not been addressed. Furthermore, the paper [27] presents a fuzzy logic-based intrusion detection model specifically designed for protecting IoT nodes with the MQTT protocol against DoS attacks. Although fuzzy logic has shown its efficiency for different applications, including sensor fault detection in IoT [28]; however, its high complexity with an increase in the input dimension limits its potential in intrusion detection for IoT where huge data are transferred continuously. In addition, more advanced and complex attacks have been left untouched in paper [27] that raises questions over the efficiency of the proposed model for detecting other types of attacks.

ML and DL has shown efficiency in detecting complex and unknown intrusions, such as MitM, DoS, etc. [23]. Commonly used algorithms include Support Vector Machine (SVM) [29,30], Semi-supervised Spatio-Temporal Deep Learning Intrusions Detection (SS-Deep-ID) [31], and Deep Feed-Forward Neural Network (DFFNN), ref. [32] etc.

In [33], multiple ML algorithms, including Autoencoder, RF, K-Means clustering, and Isolation Forest (IF), are employed to detect attacks in the IoT. However, the paper does not present clarity about the type of attacks considered in this work. In addition, the authors developed and evaluated an intrusion detection strategy on the network layer of the IoT that is not necessarily based on the MQTT messaging protocol.

Faker and Dodge [32] proposed a DL-based network intrusion detection system and evaluated it against CIC-IDS2017 and UNSW-NB15 datasets where accuracy and prediction time are used as evaluation metrics. The results show the significance of applying deep learning (DL) algorithms while designing intrusion detection systems for IoT. A total of 32 attack types from CIC-IDS2017 and UNSW-NB15 were included in the experiment using accuracy and prediction time as evaluation metrics. CIC-IDS2017 and UNSW-NB15 datasets are general purpose datasets not representing MQTT specifically. In [34], authors have worked on a new dataset known as MQTTset and proposed various ML algorithms for intrusion detection.

In [35], performances of eight different ML algorithms, including DNN, Logistic Regression (LR), NB, SVM, Adaptive Boosting (AB), kNN, DT, and RF, are analysed against six datasets, such as KDD-99, NSL-KDD, UNSW-NB15, Kyoto2006+, and WSN-DS CICIDS2017. Intuitively, the DL achieved the best accuracy as compared to classical ML classifiers at the cost of the high computational requirements. This paper also does not address MQTT messaging protocol-related issues.

Table 1 and Table 2 summarise intrusion detection systems proposed in recent literature with a tick (√) in the last column indicating the given model is developed targeting the MQTT protocol. The first four columns show the reference number for the paper, the ML model exploited the evaluation method, and the evaluation metrics, respectively.

In [24], a number of ML algorithms, such as eXtreme Gradient Boosting (XGBoost), GRUs, and LSTM, are used to design security models for the MQTT protocol in IoT. For the verification of the proposed algorithms, the author’s used the MQTT dataset containing three types of attacks such as intrusion (illegal entry), DoS, and MitM. Different ML algorithms, such as NB, RF, DT, LR, KNN and SVM, are evaluated using the MQTT-IoT-IDS2020 dataset [25]. The acceptable performance of these ML models for the proposal of the MQTT intrusion detection system was reported. The author of [40] proposed a single-layer ANN-based model for intrusion detection in an MQTT-enabled IoT system. The proposed model is evaluated on the KDD-99 and NSL-KDD dataset with acceptable performance measures. However, these datasets do not represent the MQTT-enabled IoT system-based environment. In [39], the author proposed a model for anomaly-based IDS in IoT systems using a Convolutional Neural Network (CNN) and GRUs for MQTT-IoT-IDS2020. This study presents a comparison of several ML-based models for intrusion detection in MQTT-enabled IoT systems with the proposed DNN.

## 3. Proposed Deep Neural Network (DNN) Based Intrusion Detection System

**Figure 3:**
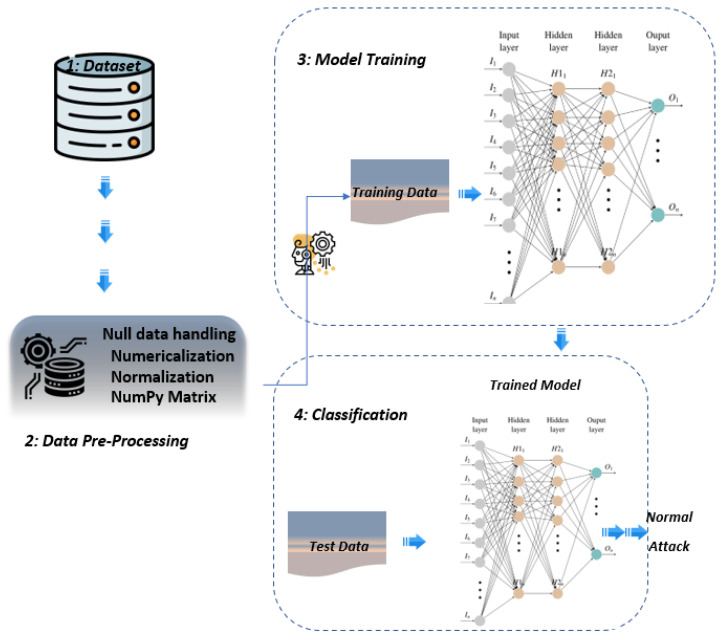
Proposed IDS framework.

Deep learning (DL) is a sub-field of machine learning inspired by the biological brain. These algorithms, also known as Artificial Neural Networks (ANNs), have better predictive capabilities as compared to conventional Multi-Layer Perceptron (MLP) because of a higher number of hidden layers. Primarily, ANNs consist of neurons connected with a neighbouring layer, which processes the input data using activation functions [41] in order to predict the output. Our proposed model consists of an input layer, two fully connected hidden layers, and an output layer. The data processing from input through the hidden layer to the output layer follows forward and backward propagation. Figure 3 shows the framework of DNN-based IDS for attack classification. The output layer is different depending upon the classification task, such as binary or multi-class. The input layer of our proposed DNN-based learning model takes into account the features of the MQTT protocol-based network, two hidden layers with Rectified Linear Unit (ReLU) activation, and an output layer with sigmoid activation in the case of binary classification and softmax for multi-class attacks classification. The reason behind choosing the softmax for multi-classification is based on our experimental results performed in this paper. As the MQTT-IoT-IDS2020 dataset contains three abstract-level features of MQTT protocol, i.e., Packet-flow, Bi-flow, and Uni-flow data. The proposed model is tested for all of the three mentioned features of MQTT contained in MQTT-IoT-IDS2020.

**Figure 4:**
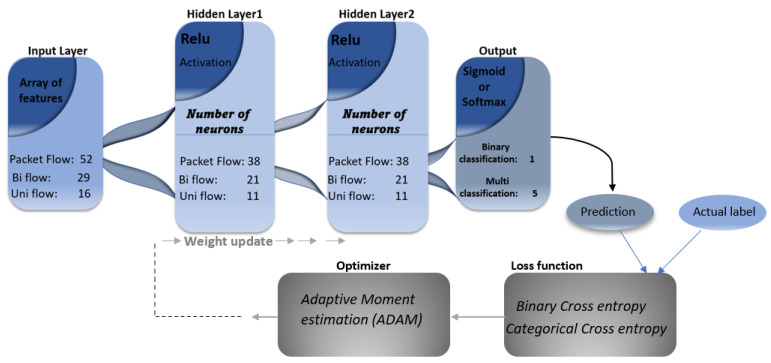
The proposed model’s training setup for binary and multi-attack classifications.

Figure 4 shows the number of input neurons, hidden layer, and output neurons. The data from the input layer is forward propagated through the hidden layer neurons during model training and backward propagated to update the weights and reduce the loss function until the model learns the proper weights and bias. Mathematically, the processing of data through the dense layer of neurons can be expressed as: $O:R^{m}\times R^{n}$, where m represents the input vector size, while n is the size of the output vector. Suppose X presents the input vector such that X = $x_{1},x_{2},x_{3}\ldots x_{m-1},x_{m}$, then the mathematical computation of the hidden layer can be expressed as a product of weights and an addition of bias as in the following equation:

(1) $$h_{i}\left(X\right)=f\left(W_{i}^{T}X+b_{i}\right)$$

where $h_{i}$ is defined as $h_{i}:R^{d_{i}-1}\to R^{d_{i}}$, f is function from f:R→R defined by (2a) for the hidden layer. In Equation (1), the $b_{i}\in R^{d_{i}}$ presents the bias that add to the product input and weights, i.e., $W_{i}\in R^{d\times d_{i}-1}$.

(2a) $$\begin{array}{c} ReLU=max(0,x) \end{array}$$

(2b) $$\begin{array}{c} Softmax\left(x_{i}\right)=\frac{e^{x_{i}}}{\sum_{1}^{n}e^{x_{j}}} \end{array}$$

(2c) $$\begin{array}{c} Sigmoid=\frac{1}{1+e^{-x}} \end{array}$$

An Artificial Neural Network (ANN) consists of many stacked hidden layers that become a deep network. In general, these hidden layers can be expressed mathematically via Equation (3).

(3) $$H\left(X\right)=H_{l}(H_{l-1}\left(H_{l-2}\left(...\left(H_{1}\left(X\right)\right)\right)\right)$$

Our proposed model is tested for binary as well as multi-class attack classification. Therefore, two different activation functions at the output layer are used. For binary classification, $\hat{y}$ is calculated at the last layer via sigmoid, as presented with mathematical expression in Equation (2c). Depending on the classification task, we utilised different cost functions; for binary attack classification, we used binary cross-entropy, as presented in Equation (4), while in the case of multi-attack classification, we utilised categorical cross-entropy, as presented in Equation (5). The loss function calculated the amount of difference between predicted labels and actual labels. The smaller the reduction in the loss function, the more accurate the prediction of the model. Optimisation algorithms play the main role in finding parameters in order to minimise or maximise any mathematical functions. In deep learning, such optimisation algorithms helps to reduce the cost function for particular. Out of many existing optimisation algorithms used in deep learning, we adopted Adaptive Moment Estimation (ADAM) as an optimiser to reduce the cost function of our proposed model. The ADAM optimiser combines the best feature Root Mean Square Propagation (RMSProp) optimiser and momentum. That is why it is still the best optimiser in most DL-related tasks and is used in lots of optimisation problems in deep learning function:

(4) $$J\left(y,\hat{y}\right)=\frac{1}{t}\sum_{i=1}^{t}-\left(y_{i}\times \log_{}\left(\hat{y}\right)+\left(1-y\right)\times \log_{}\left(1-\hat{y}\right)\right)$$

(5) $$J\left(y,\hat{y}\right)=\frac{1}{t}\sum_{i}^{t}\sum_{j}^{c}y_{ij}\log_{}\left(\hat{y}\right)$$

where *J* is a function defined on *y* and $\hat{y}$, $\hat{y}$ is predicted output calculated at the last layer by sigmoid or softmax of our proposed model, and *y* is the actual label, *t* is the batch size, and *c* denotes the class category.

### 3.1. Other Classical ML Models

This subsection highlights the brief theoretical concepts behind the other classical ML models that are used for cross-comparison in this study with the proposed Deep Neural Network model.

#### 3.1.1. K-Nearest Neighbour

This learning algorithm is categorised as a supervised learning model and known as a lazy learner because of the fact that it does not learn a discriminative function from the training data rather memorise it. For example, the weights during the training process of the logistic regression are learned. The KNN algorithm is relatively straightforward, the working of KNN can be summarised in the following three main points:

- Choose the number of *k* neighbour and distance metrics.
- Locate the *k* neighbour of the test sample.
- Assign label accordingly to the majority of the label in *k* neighbour.

Different distance metrics exist such as Manhattan distance, Minkowski distance, and Euclidean distance, etc. Among all of these, the euclidean is widely used as a distance metric in KNN. The Euclidean distance and Manhattan distance is a specialised form of Minkowski. The mathematical representation of these distances is given as below in Equation (6).

(6) $$d\left(x^{i},x^{j}\right)=\sqrt[{p}]{\sum_{k}{\left|x_{k}^{i}\;\;\;x_{k}^{j}\right|}^{p}}$$

where the parameter p, if changes then the above equation change to other distance metrics. For example, if p=2 then the above equation becomes euclidean, and if p=1 then it becomes Manhattan distance.

#### 3.1.2. Decision Tree

This model breaks our data into a hierarchical manner, so that to make predictions on new data, that is why due to this hierarchical learning style of this model it is called a decision tree. This learning model also belongs to supervised learning and can handle both classification & regression problem. This model makes tree, where each node of the decision tree model represents an attribute and each leaf node represents a class label. The main working of the decision tree can be described as:

- Find the best attribute and place it in the root of the tree.
- Make subset of training data in such a way that each subset contains data with the same value for an attribute.
- Repeat above two steps until reach to the leaf node.

Assume a dataset consist of n attributes, for the selection of best attribute as the root node of the tree, researcher work on mathematical measures, these mathematical measured values are used for such attribute selection, these measures are information gain and Gini-index. Mostly the information gain is used when the attribute is categorical, while for continuous attributes the Gini index is favourable. An information gain is a reduction in entropy. Entropy is a measure used to calculates the randomness of data within attributes or features of a dataset. Mathematically entropy can be represented as bellow equation

(7) $$Entropy=\sum_{i=1}^{c}-p_{i}*log_{2}\left(p_{i}\right)$$

here $p_{i}$ denotes the proportion of the sample that belongs to class *c*. The following steps are used in calculating the information gain using entropy.

- Calculates the entropy of the target attribute.
- Calculates the entropy of other attributes and subtract from the entropy of target.

Mathematically it is represented as:

(8) InformationGain=E(Target)−E(Target,Attribute)

#### 3.1.3. Random Forest

Random Forest learning model is a type of supervised ML model. It is an ensemble model which makes use of multiple trees in predictions of a target. This model is used for regression and classification problems. It takes n samples as input and creates multiple trees based on a subset of input features. Then on the results of every tree, a majority voting is performed in order to get the final prediction for the target class variable.

Assume m denotes the total features in data, the main working of this learning model can be summarised in the following points.

- Select *k* number of features randomly from m features of data such that *k* < < m.
- Calculates the best split for k selected features.
- Split the node into child nodes using best split.
- Repeat above until leaf node reached.
- Build a forest of trees by repeating the above steps.

#### 3.1.4. Naive Bayes

This learning model is based on Bayes rules in learning and predicting the new instances class label. Bayes theorem provides the way of calculating the posterior probability of class as depicted in Equation (9) below.

(9) $$p\left(c|x\right)=\frac{p(x|c)\;\;p(c)}{p(x)}$$

where p(c∣x) indicates the posterior probability of the target class given independent variable *x*, p(c) indicates the prior probability of the target class, p(x∣c) represents the likelihood and p(x) is the prior probability of independent variable. In comparison to other, the NB performs better and fast prediction of the test set. This model performs better in multi-classification problems. sci kit-learn provides three types of models for Naive Bayes, these are Gaussian, Multinomial, Bernoulli.

## 4. Experimental Setup and Results

### 4.1. Dataset Selection

Dataset helps in evaluation of ML model performance against attack detection for the network.

Numerous datasets for the evaluation of IDS are proposed by researchers in the area of network security. That helps in understanding the performance of particular machine learning or deep learning algorithms for intrusion detection. We reviewed the existing publicly available dataset for IDS evaluation in order to find the best representative for our proposed IDS evaluation in MQTT enables IoT systems. There are two types of datasets available in network security. Table 3 compares the existing datasets available for evaluations and the representation of each dataset for MQTT protocol, whether a dataset is a general-purpose or special purpose.

#### 4.1.1. General Purpose

The general-purpose datasets are referred to as those which are generally used for IDS evaluation representing a general computer network. They are not created for specific networks nor replicating the specific types of IoT protocols. For example, Canadian Institute for Cybersecurity proposed several general-purpose NIDS datasets such as CIC-DoS dataset [49], CIC-IDS2017 [49] and CIC-IDS2018 [50], which helps the researchers community in intrusion detection to test the performance of ML or DL based learning models. In the literature, there are multiple datasets for general-purpose Network Intrusion Detection System (NIDS) evaluation, most common of them used for example KDD-99 [49], NSL-KDD [49] etc. The researcher evaluates the performance of the proposed IDS by using such a dataset for generalised networks. A lot of research exists over these datasets, that shows the significance of each ML, DL, or Ensemble Learning (EL).

#### 4.1.2. Special Purpose

The dataset was created for special purposes to represent specific types of networks or protocols. There are some specialised networks, such as IoT-based [51], Internet of Vehicles (IoV) networks [52], and Supervisory Control and Data Acquisition (SCADA) network [53]. These networks are comprised of some specialised devices that can not be found in other networks. These devices are especially dedicated to such networks, for example, remote terminal units [54] in SCADA networks, etc. Table 2 shows the special and general-purpose datasets along with evaluated algorithms of very recent research. After our review, we selected the MQTT-IoT-IDS2020 dataset to check the performance of our proposed algorithm for the MQTT protocol. However, this table clearly shows that a Deep Neural Network for MQTT protocol is still an interesting idea. The focus of this work is to evaluate the Deep Neural Network for the MQTT protocol.

#### 4.1.3. Prepossessing and Description of Selected Datasets

For our proposed IDS, we chose MQTT-IoT-IDS2020 [25] and the other latest published MQTT dataset (https://joseaveleira.es/dataset; access date was 8 July 2021) [24] for testing the performance of the proposed DNN algorithm. There were sensors, a camera, and other devices that communicate with each other via the MQTT protocol while capturing these datasets. Table 4 and Table 5 show the statistics of these two datasets used in the current study for the performance evaluation of the proposed IDS. These are the latest datasets and are created in an MQTT protocol-enabled simulated environment; hence, they better represent the features of the MQTT protocol-based IoT network.

**Figure 5:**
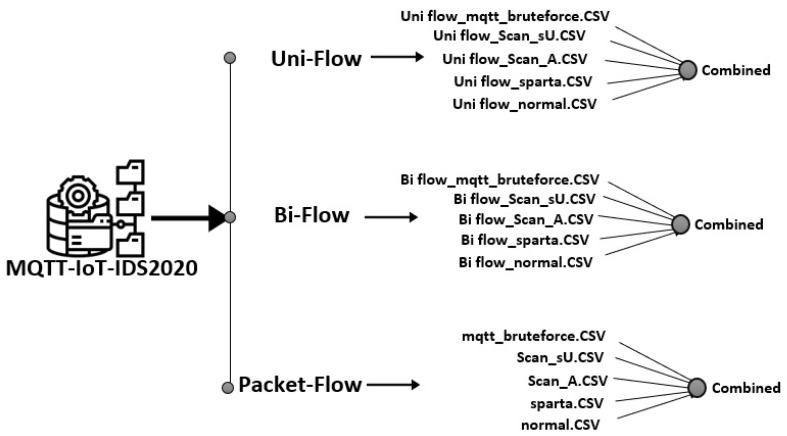
MQTT-IoT-IDS2020 pre-processing.

**Figure 6:**
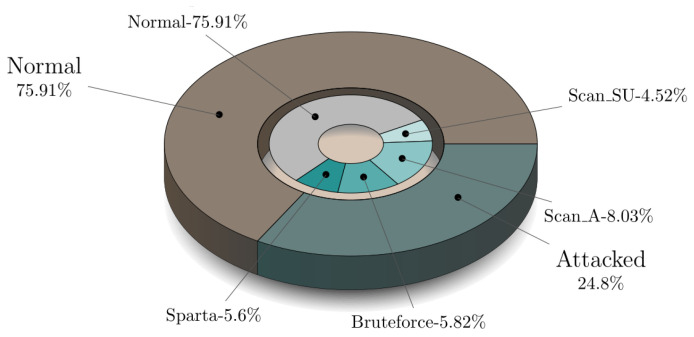
Five files statistics of Uni-flow.

**Figure 7:**
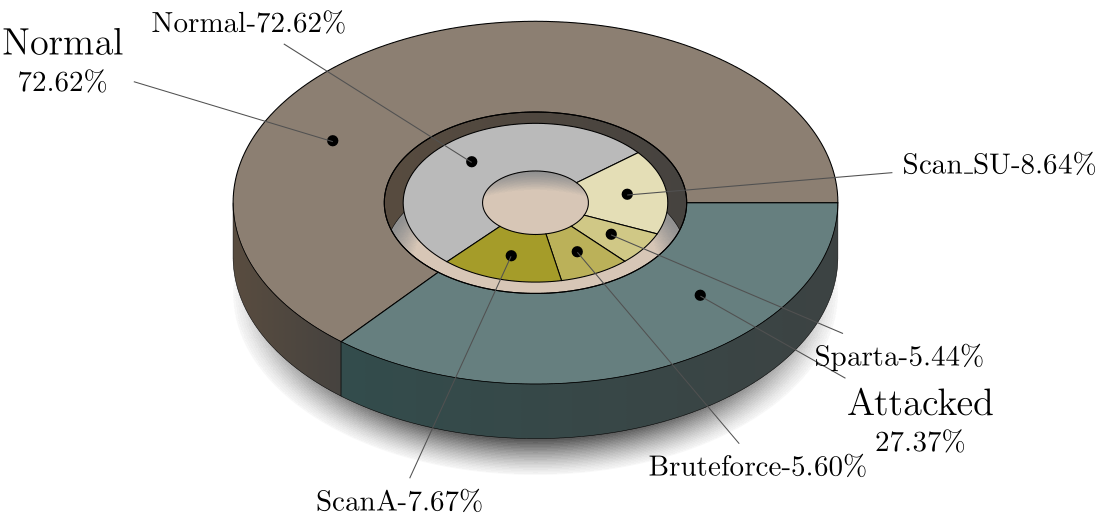
Five files statistics of Bi-flow.

**Figure 8:**
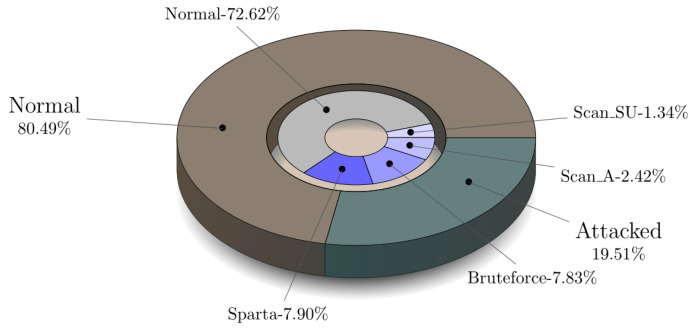
Five files statistics of Packet-flow.

Five scenarios were launched during the creation of the MQTT-IoT-IDS2020 dataset. These scenarios are normal operation, aggressive scan, UDP scan, Sparta SSH brute-force, and MQTT brute-force attack [25]. Each of these scenarios is recorded in separate files for three abstraction level network flow features of MQTT enabled simulated network. These flow features include Packet-flow, Uni-flow, and Bi-flow features. Every flow-level features of MQTT have five files representing attack and normal of particular scenario as mentioned above. For all of these Packet-flow, Bi-flow, Uni-flow data, we implemented a python script to combine all of these five files in each network flow-level into one combined CSV and create a combined dataset for each network flow-level feature. The combined CSV contains the binary label and multi-class label attribute in order to test the proposed algorithm performance over the MQTT protocol recorded traffic for binary as well as multi-attack classification. Figure 5 presents files of MQTT-IoT-IDS2020 in each network flow feature and their combined version dataset for each flow-level features of MQTT-IoT-IDS2020. For example, in Uni-flow feature data of MQTT, there are five files, and we combined all of these five files into one CSV with two extra columns with attack and the type of attack. The statistic of the combined version data for Packet-flow, Bi-flow, and Uni-flow is given in Figure 6, Figure 7 and Figure 8 in the form of nested pie plot. The most outer plot shows the normal and attacked instances, and the inner pie plot shows the statistics of each of the five scenarios separately presenting the distribution of multi-type attacks. The reason behind merging and creating binary as well as multi-class combined version datasets is to test the proposed IDS performance on binary and multi-class attack classification for each type of network features, i.e., Packet-flow, Bi-flow, and Uni-flow [25]. The final prepossessed dataset is split into two parts: 80% for training and 20% for testing the trained ML model performance.

In order to test the performance of the proposed DNN approach for IDS in an MQTT-based IoT system, we performed some pre-processing to the combined version of each feature level data in order to further prepare it. Because ML/ DL algorithms have requirements before applying these algorithms to data. All the features explanations of the dataset are available in detail in [25]. We analysed the data in each network flow-level features data of MQTT-IoT-IDS2020, i.e., Uni-flow, Bi-flow, and Packet-flow. As discussed above and mentioned in Figure 5, for each flow-level feature, all the attacked and normal scenarios were combined into one combined.csv file. Among all these, the Packet-flow features have some attributes that contained nulls value above 90 percent; these attributes are mqtt_flag_uname, mqtt_flag_passwd, mqtt_flag_retain, mqtt_flag_qos, mqtt_flag_willflag, mqtt_flag_clean, and mqtt_flag_reserved. We removed such attributes by threshold. For the remaining features in packet featured data, we put the median to fill the null-valued variable as the median is less susceptible to an outlier. Apart from this, we perform label encoding and one-hot label encoding as all TCP and IP flags were label encoded, and we perform one-hot encoding to express protocol features more clearly to our proposed model, due to which the packet feature increased as there were different protocols in the dataset. In Uni-flow and Bi-flow featured data, we removed certain features, such as source, destination IP address, and time stamp, etc. Besides this, certain features, such as MQTT flags, were removed in [25]; however, we have not removed such categorical features, but we instead prepared it by numerically encoding by the label encoder. Some features contained multi-type data, for example, string and int64 in one feature, so we converted the string into int64 representation in order to prepare for deep learning model. Features such as tcp_flag_res, tcp_flag_ns, tcp_flag_ecn, and protocols are converted, as and we performed label encoding as they are categorical in nature. Apart from this, we performed feature scaling to some features in Packet, Uni-flow, and Bi-flow data. Features such as mean_pkt_len, num_byte, min_pkt_len, max_pkt_len in Uniflow; fwd_num_byte, fwd_num_byte, fwd_max_pkt_len in Bi-flow; and ip_len in Packet-flow have scaled using feature scaling in python.

### 4.2. Experimental Setups

#### 4.2.1. Evaluation Metrics

Understanding the model performance requires the statistical ground truth values, which measure how the model performs in attack classification from normal data. Several evaluation metrics can be used to check the performance of an IDS. These metrics are shown from Equation (10a–d).

(10a) $$\begin{array}{c} \mathrm{Accuracy}=\frac{TP+TN}{TP+TN+FP+FN} \end{array}$$

(10b) $$\begin{array}{c} \mathrm{Precision}=\frac{TP}{TP+FP} \end{array}$$

(10c) $$\begin{array}{c} \mathrm{Recall}=\frac{TP}{TP+FN} \end{array}$$

(10d) $$\begin{array}{c} F1-measure=2\times \left(\frac{Precision\times Recall}{Precision+Recall}\right) \end{array}$$

where:

- *TP* is the correct classification of normal as normal flow.
- *TN* is the correct classification of attacked as attacked flow.
- *FP* is the incorrect classification of normal as attacked flow.
- *FN* is the incorrect classification of attacked as normal flow.

Accuracy is the ratio of the correctly classified connection records to the complete test dataset. Machine learning (ML) or deep learning (DL) algorithms are considered to be the best model if their accuracy is high. Precision refers to the ratio of the accurate detection of attacked instances to the number of all detected attacked instances. This is the second measure for the evaluation of the machine learning algorithm: if the model is categorised high, it is a good model. A recall is another measure used to evaluate the ML algorithm performance that gives the relation between true positive ( *TP*) predictions to true positive ( *TP*) and false negative ( *FP*) predictions. In calculating the F1 measure, the precision and recall are both used as a harmonic mean.

#### 4.2.2. Programming Libraries and Parameters Setup

Keras (the Python deep learning API) with a hardware specifications Core i7 processor (16 GB RAM) is used in this work. The experiment was implemented in python 3.9.5 programming language using an interactive Python-based IDE named Jupyter notebook inside Anaconda distribution used as a software tool for the implementation and evaluation of proposed experiments. We used the pandas-profiling 2.11.0, which is an open-source module of python that provides the facility for data analysis. There are various things that have an effect on the results of the DL algorithm, for example, batch size, learning rate, type of optimiser for reduction of the loss function. The optimiser in designing the deep learning algorithms plays the main role because it reduces the cost function with less effort and less resource usage, but it depends on the nature of the optimiser. Accuracy increases as the reduction in the loss function. For the reduction in the loss function in our proposed model, i.e., binary-cross entropy or categorical-cross entropy, we studied and reviewed the existing optimiser, and we select the ADAM [55] optimiser out of the existing available optimisers because it will optimise the categorical cross-entropy loss function in the case of multi-classification, while binary-cross entropy will be optimised via ADAM in the case of binary classification in the proposed model.

ADAM combines the advantages of two Stochastic Gradient Descent (SGD) extensions, i.e., Adaptive Gradient Algorithm (Adagrad) [56] and Root Mean Square Propagation (RMSProp) [57]. ADAM implements momentum that brings smoothing and fast searching during training [57], and with the help of RMSProp optimiser, they change the learning rate efficiently during training time [57], which converges very quickly towards the global minima. Due to both of the mentioned advantages, we choose the ADAM as an optimiser in our proposed model. Certain studies have revealed that the learning rate and batch size have high correlations between each other [58,59,60], which is if there is a change in learning rate or in batch size, then the accuracy is impacted by such changes. In [58], it is clearly showed that when the learning rate is small, with the increase in batch size, the accuracy slows down, and while increasing the batch size with a slightly large learning rate, the accuracy increases. Therefore, we run multiple experiments to find the optimal batch size of our final model for binary as well as multi-attack classifiers. We summarised all of the results of different batch sizes, as shown in Table 6, Table 7 and Table 8. They also show a comparison among batch size performance of the classification MQTT. Out of our experiment with different batch sizes and different activation functions in the last layer that has the same hidden layer activation (relu) and network architecture, the higher batch for all three abstraction level features is recommended.

We tested the 32, 64, 128, and different activation functions in the last layer for binary as well as multi-attack classification. As the batch size increases, the accuracy increases. Out of all the experiments, the batch size of 128 proved optimal and gave good results in comparison to other lower batches. Based on the results in Table 6, Table 7 and Table 8, we recommend a higher batch size for the MQTT-IoT-IDS2020 dataset. Another advantage with the higher batch size is the number of iterations in each epoch decreases, which saves much of the learning time, and the model runs efficiently and fast.

### 4.3. Performance Analysis and Discussion

**Figure 9:**
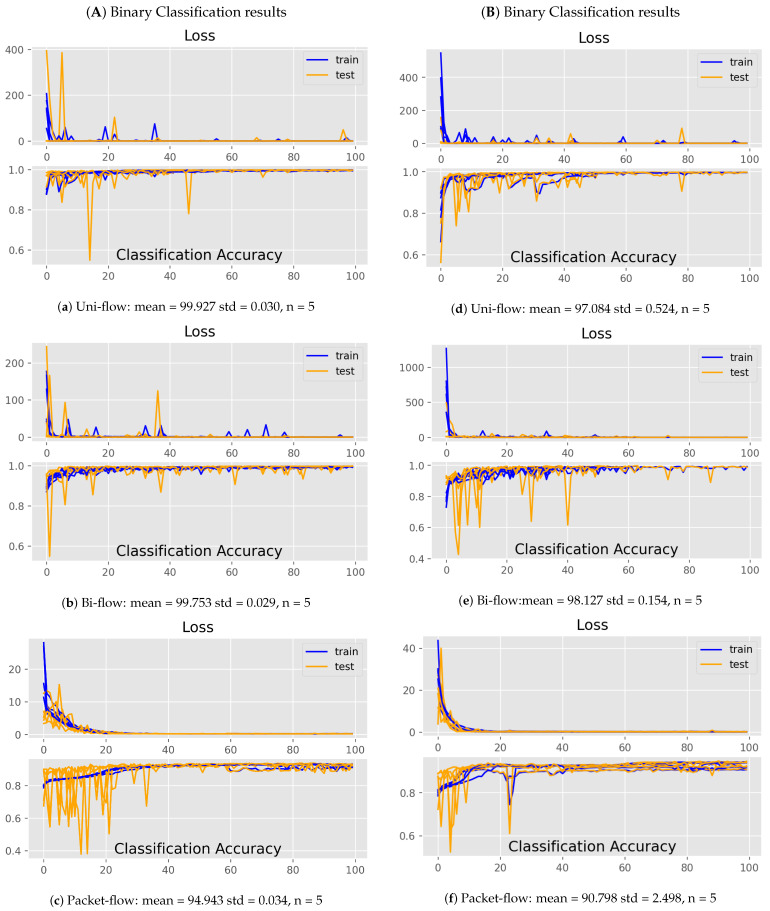
Five-fold cross validated accuracies for 3 abstraction-level features of MQTT.

This section discusses the results obtained. Based on the experiments of Table 6, Table 7 and Table 8, we selected 128 batch size with a sigmoid output function at the last layer for binary classification, softmax for multi-classification, and the ADAM optimiser, which will optimise our cost function for binary classification as well as multi-classification of an attack for our proposed deep learning model toward intrusion detection in MQTT protocol-based smart IoT systems. Choosing a higher batch size reduces the iteration in each epoch, which saves a lot of time during model training. Due to the stochastic nature of the proposed algorithms, the results may vary each time the model runs because the weights are randomly assigned during training the model each time. Every time the model runs, it learns differently because of its stochastic nature. Furthermore, we performed a five-fold cross-validation evaluation on the proposed model to find the average evaluation metrics, i.e., accuracy, precision, recall, etc., with standard deviation. Table 9 and Table 10 show the five-fold cross-validated evaluation accuracy of our proposed model against binary as well as multi-class attack classification, respectively. For binary classification, the proposed model performed well on both Bi-flow and Uni-flow features data of MQTT with 99.753 and 99.927 mean accuracy, respectively. While in the case of Packet-flow data of MQTT, it performed with a mean accuracy equal to 94.943. Figure 9a–f shows learning curves of the five-fold binary attack classification and multi-attack classification in terms of loss and accuracy for each abstraction level of the feature of MQTT, i.e., Uni-flow, Bi-flow, and Packet flow.

The quick convergence of the model can be observed. Learning curves help in understanding and diagnosing an over-fit, under-fit, or well-fit model on the training and testing datasets. A model is said to be under-fitted in two cases identified from the learning curve: in case 1, the training loss curve will form a straight line, i.e., no change, and in case 2, the model loss will continuously decrease at the end of the model training process. While in over-fitting, the testing loss begins to increase after some time faster than the training loss curve. The well-fit model shows a different learning curve than over-fitted and under-fitted models. That is, a model is said to be well-fit if the training and testing curves have a small gap or it decreases to a point of stability. Similarly, as mentioned above, we can understand the model performance on the MQTT-IoT-IDS2020 dataset by observing Figure 9a–f. Packet-flow accuracy is less than the others, i.e., Uni-flow and Bi-flow. One of the reasons for low accuracy on Packet-flow feature data of MQTT is the imbalance of the attack classes’ distribution of Packet-flow data in each file. In order to reduce the imbalanced class problem, we have selected the data from five files of Packet-flow feature data of the MQTT-IoT-IDS2020 dataset. We further evaluate our model in order to test its performance on other evaluation metrics, such as precision, recall, and F1-measure. Table 11, Table 12 and Table 13 show the binary classification of our proposed model in terms of precision, recall, and F1-measure, respectively. Table 14, Table 15 and Table 16 show the multi-attack classification evaluation in terms of precision, recall, and F1-measure. To further test the suitability of the proposed model for intrusion detection in MQTT-based smart IoT systems, we compared its performance with other classical machine learning models, such as DT, RF, NB, and KNN for binary and multi-class classification.

Table 17 and Table 18 show the comparison of the proposed model with other classical machine learning models. The comparison was carried out using a number of parameters, such as accuracy, precision, recall, F1 measure, and training and testing times. The training and testing time in Table 17 and Table 18 are measured in seconds. Depending on the specification of the system, the training and testing times may vary (we have already previously mentioned the system specifications). All of the classical machine learning algorithms mentioned in Table 15 and Table 16 perform well on Uni-flow and Bi-flow data of MQTT protocol. However, on the Packet feature of MQTT data, our proposed model, in most cases, provides better results than other classical machine learning models. We also tested and compared the performance of our proposed model with other deep learning models’, such as GRUs and LSTM, performance against MitM, Intrusion, and DoS over MQTT-enabled IoT network. One can see from Table 19 that the accuracy and F1-score of the proposed model are higher than other traditional learning models.

## 5. Conclusions and Future Direction

This paper presents a DNN-based intrusion detection system for MQTT-enabled IoT smart systems. A recently published MQTT-IoT-IDS2020 and another MQTT dataset are used to evaluate the performance of the proposed model. The MQTT-IoT-IDS2020 dataset contains three abstraction-level features of MQTT-enabled IoT, including Packet-flow, Bi-flow, and Uni-flow features. There are five files in each of these featured data representing attack and normal scenarios. The data were organised such that each separated feature gets a subset in order to assess the performance in binary-class and multi-class attack classification. The tests were conducted under different batch sizes, such as 32, 64, and 128, for binary and multi-classifications. The results show that increasing the batch size of the training subset improves the convergence and performance of the classifier. The performance of the proposed DL-based IDS with a default learning rate and using the ADAM optimiser was compared with the performance of conventional ML-based IDSs, including KNN, NB, DT, and RF. Furthermore, the proposed model was tested for binary-class as well as multi-class attack classification with different activation functions at the output layers. The results show that the DL-based model for Bi-flow and Uni-flow featured data can achieve 99% accuracy and 98% accuracy for binary and multi-class attack classification, respectively. However, in Packet-flow featured data, the accuracy for binary and multi-class were 94% and 90%, respectively. Additionally, we also tested the performance of the proposed model against DoS and MitM, etc., over an MQTT-based IoT system. From the results and comparison tables, it was evident that the proposed model has higher accuracy than other state-of-the-art deep learning models. In the future, we intend to investigate the vulnerability of new types of attacks on various IoT protocols. Our aim is to propose a novel deep learning-based model for new vulnerabilities.

## Figures and Tables

**Table 1:** Intrusion detection systems for MQTT protocol.

Paper Ref	Method Used	Evaluation Method	Evaluation Metrics	MQTT Protocol
[26]	Process Tree	Network modeling	Detection rate	√
[27]			Detection efficiency	
	Fuzzy Logic	Network traffic	Detection rate	√
			Detection accuracy	
			False positive ratio	
[31]	Deep Learning(DL)	CIC-IDS2017	Accuracy, Precision	✕
		CIC-IDS2018	Recall, F1 score	
[25]	Gaussian NB		Accuracy	√
	LR		Precision	
	KNN	MQTT-IoT-IDS2020	Recall	
	SVM		F1-score	
	DT			
	RF			
[34]	RF		Accuracy	
	NB, Neural network		F1-score	√
	DT	MQTTset	Confusion matrix	
	Gradient boost			
	Multi-Layer Perceptron (MLP)			
[35]	NB, SVM	UNSW-NB15	Accuracy	
	Adaptive Boosting	Kyoto2006+	Precision	
	KNN	WSN-DS	Recall	✕
	DT	CICIDS2017	F1-score	
	RF		RoC, AUC	
[36]	NB, SVM			
	DT	ADFA-LD	Precision	✕
	SVM-RBM, GRU	KDD-99	Recall	
	Text-CNN		F1-score	
[37]	CNN-LSTM, BLSTM	CIDDS-001	Accuracy	
	Ensemble, DNN	UNSWNB15	Specificity	✕
	MLP	KDD-99	Sensitivity	
	DT			

**Table 2:** Review of Intrusion detection systems for MQTT protocol.

Paper Ref	Method Used	Evaluation Method	Evaluation Metrics	MQTT Protocol
[24]	XGBoost		Log loss	√
	GRUs	Intrusion dataset	Cross entropy	
	Neural network		Accuracy	
	LSTM		F-beta score	
[38]	31 ML algorithms		Accuracy	
	supervised and	CICIDS2017	precision and recall	
	Unsupervised		F-beta score	✕
			True positive	
			negative rate	
[32]	Deep Learning	CICIDS2017	Accuracy	✕
	Gradient boosting	UNSW-NB15	prediction time	
	Random Forest			
[39]	CNN–GRUs	MQTT-IoT-IDS2020	Accuracy	
		BoT-IoT	precision and recall	
			F-score	✕
			Accuracy	
Proposed	DNN	MQTT-IoT-IDS2020	Precision	√
		Intrusion dataset	Recall	
			F1-measure	

**Table 3:** Special and general purposed dataset for IDS evaluation.

Dataset Reference	General Purpose	Special Purpose	MQTT Protocol	Algorithms Evaluated	Paper References
CIC-IDS2017				RF, SVM, DT	
Kyoto	√	✕	✕	DNN, NB, kNN	[35]
WSN-DS				LR, AB	
UNSW-NB					
KDD-99					
				GA	
NSL-KDD	√	✕	✕	RF, SVM, DT	[42]
				Kernel ELM, kNN	
				NB, MLP	
				DNN	[43]
KDD-99	√	✕	✕	NB, DT, RF	[44]
				ontology	
				RF, SVM, DT	
				DNN, NB, kNN	[35]
				LR	
3				DFFNN	[32]
CICIDS2017	√	✕	✕	GBT	
				RF, SVM, DT	
				DNN, NB, kNN	[45]
				LR, AB	
Bot-IoT	✕	√	✕	SNN	[46]
Ton-IoT	✕	√	✕	NB, SVM, RF, kNN	[47]
				DT, LR, LSTM, LDA	
MQTT-IoT-IDS2020	✕	√	√	NB, SVM, RF, kNN	
				DT, LR	[25]
(DoS)					
(Man in the middle)	✕	√	√	XGBoost, LSTM	[24]
(Intrusion) [48]				GRU, NN	
MQTTset	✕	√	√	NN, RF, MLP	
				DT, GBT	[34]

**Table 4:** Three abstract-level feature statistics of MQTT-IoT-IDS2020.

	Classes	Total Instances	Normal	Attack
Bi-flow	Biflow_mqtt_bruteforce	16,696	2152	14,544
	Biflow_normal	86,008	86,008	✕
	Biflow_scan_A	25,693	5786	19,907
	Biflow_scan_sU	39,664	17,230	22,434
	Biflow_sparta	91,318	77,202	14,116
Uni-flow	Uniflow_mqtt_bruteforce	33,079	4205	28,874
	Uniflow_normal	171,836	171,836	✕
	Uniflow_scan_A	51,358	11,561	39,797
	Uniflow_scan_sU	25,845	34,409	22,436
	Uniflow_sparta	182,407	154,175	28,232
Packet-flow	mqtt_bruteforce	90,876,584	70,980,732	19,895,852
	Normal	1,056,230	1,056,230	✕
	scan_A	111,392	70,768	40,624
	Biflow_scan_sU	233,255	210,819	22,436
	Biflow_sparta	130,876,584	90,980,732	39,895,852

**Table 5:** MQTT dataset ^1^ statistics.

File Name	Total	Normal	Attacked
DoS.CSV	94,625	49,112	45,513
Intrusion.CSV	80,893	78,995	1898
MitM.CSV	110,668	106,813	3855

^1^https://joseaveleira.es/dataset.

**Table 6:** Binary-class attack classification with the sigmoid activation function in the output layer.

Network Feature	Batch Size	Training Accuracy	Testing Accuracy
Packet-flow	32	93.74	92.93
	64	93.43	93.43
	128	94.99	94.57
Bi-flow	32	99.32	99.29
	64	99.59	99.66
	128	99.99	99.92
Uni-flow	32	99.73	99.74
	64	99.63	99.77
	128	99.94	99.98

**Table 7:** Multi-class attacks classification with sigmoid activation function on output layer.

Network Feature	Batch Size	Training Accuracy	Testing Accuracy
Packet-flow	32	88.66	88.62
	64	88.71	88.78
	128	89.51	89.71
Bi-flow	32	94.32	93.89
	64	93.59	94.06
	128	93.99	93.92
Uni-flow	32	94.13	93.99
	64	93.83	93.77
	128	94.04	93.98

**Table 8:** Multi-class attacks classification with softmax activation function on output layer.

Network Feature	Batch Size	Training Accuracy	Testing Accuracy
Packet-flow	32	90.02	90.17
	64	90.71	90.78
	128	91.71	91.96
Bi-flow	32	97.32	98.89
	64	97.59	97.99
	128	98.02	98.34
Uni-flow	32	96.99	97.39
	64	97.97	97.87
	128	97.04	97.91

**Table 9:** Binary-class attack classification with five-fold cross validation results in terms of accuracy.

Network Features	Accuracy	Mean	Standard Deviation
Packet-flow	94.996	94.943	0.034
	94.907		
	94.936		
	94.966		
	94.911		
Bi-flow	99.788	99.753	0.029
	99.730		
	99.711		
	99.778		
	99.759		
Uni-flow	99.9333	99.927	0.030
	99.929		
	99.950		
	99.869		
	99.953		

**Table 10:** Multi-class attacks classification results with five-fold cross validation in terms of accuracy.

Network Features	Accuracy	Mean	Standard Deviation
Packet-flow	91.900	90.798	2.498
	91.022		
	85.932		
	92.524		
	92.615		
Bi-flow	98.063	98.127	0.154
	97.966		
	98.340		
	98.275		
	97.993		
Uni-flow	96.872	97.084	0.524
	96.916		
	97.549		
	96.306		
	97.779		

**Table 11:** Binary-class attack classification results in terms of precision.

Network Feature	Precision	Mean	Std
Packet-flow	93.833	93.853	0.099
	93.752		
	93.638		
	93.943		
	94.001		
Bi-flow	99.911	99.881	0.021
	99.857		
	99.873		
	99.863		
	99.902		
Uni-flow	99.986	99.953	0.034
	99.914		
	99.986		
	99.972		
	99.910		

**Table 12:** Binary-class attack classification results in terms of recall.

Network Feature	Recall	Mean	Std
Packet-flow	86.733	86.815	0.119
	86.852		
	86.648		
	86.843		
	87.001		
Bi-flow	88.711	88.699	0.015
	88.697		
	88.693		
	88.720		
	88.675		
Uni-flow	87.996	88.064	0.058
	88.110		
	88.105		
	87.991		
	88.120		

**Table 13:** Binary-class attack classification results in term of F1-score.

Network Feature	F1 Score	Mean	Std
Packet-flow	90.143	90.188	0.118
	90.170		
	90.007		
	90.253		
	90.365		
Bi-flow	93.978	93.958	0.011
	93.946		
	93.951		
	93.962		
	93.954		
Uni-flow	93.609	93.633	0.025
	93.641		
	93.670		
	93.600		
	93.645		

**Table 14:** Multi-class attacks classification results in term of precision.

Network Feature	Precision	Mean	Std
Packet-flow	89.693	89.4118	1.115
	90.970		
	88.187		
	88.099		
	90.110		
Bi-flow	95.378	95.109	0.142
	95.116		
	94.991		
	94.988		
	95.074		
Uni-flow	94.654	94.761	0.535
	94.641		
	95.670		
	93.998		
	94.845		

**Table 15:** Multi-class attacks classification results in term of recall.

Network Feature	Recall	Mean	Std
Packet-flow	81.385	81.640	0.403
	81.970		
	82.107		
	80.999		
	81.743		
Bi-flow	86.331	86.717	0.433
	87.116		
	86.591		
	86.222		
	87.324		
Uni-flow	85.966	86.435	0.421
	86.817		
	86.398		
	87.002		
	85.990		

**Table 16:** Multi-class attacks classification results in term of F1-score.

Network Feature	F1 Score	Mean	Std
Packet-flow	85.337	85.346	0.620
	86.235		
	85.038		
	84.399		
	85.722		
Bi-flow	90.629	90.718	0.235
	90.940		
	90.596		
	90.393		
	91.034		
Uni-Flow	90.101	90.605	0.375
	90.560		
	90.798		
	90.364		
	90.201		

**Table 17:** Performance comparison of the proposed DL-based IDS with other ML algorithm-based IDSs (binary-class classification).

Dataset	Models	Accuracy	Precision	Recall	F1 Score	Training Time (s)	Testing Time (s)
Packet-flow	K-NN	83.980	80.643	78.654	79.636	63.33	14.47
	NB	49.780	45.503	50.003	47.646	7.70	4.41
	DT	90.970	91.211	89.108	90.147	9.10	4.18
	RF	89.985	90.345	88.976	89.655	10.37	4.65
	Proposed	94.943	93.853	86.815	90.188	195.83	6.10
Bi-flow	K-NN	98.999	99.673	81.991	89.971	53.77	12.30
	NB	94.188	94.007	80.693	86.843	5.80	3.20
	DT	98.698	99.467	84.377	91.303	7.80	3.15
	RF	99.537	99.631	84.117	91.219	8.10	3.35
	Proposed	99.753	99.881	88.699	93.958	168.83	4.70
Uni-flow	K-NN	99.125	99.116	98.023	98.566	48.88	11.55
	NB	90.065	88.213	70.003	78.060	6.92	3.90
	DT	99.111	99.345	97.932	98.633	7.39	3.05
	RF	99.061	99.111	97.999	98.551	8.51	3.83
	Proposed	99.144	98.395	98.984	98.688	145.68	4.90

**Table 18:** Performance comparison of proposed DL-based IDS with other ML algorithm-based IDSs (multi-class classification).

Dataset	Models	Accuracy	Precision	Recall	F1 Score	Training Time (s)	Testing Time (s)
Packet-flow	K-NN	81.333	78.993	77.991	78.489	61.74	13.90
	NB	48.786	70.332	49.156	57.868	7.20	3.90
	DT	90.539	89.832	79.195	84.178	8.90	4.37
	RF	89.865	88.991	81.327	84.987	9.30	3.98
	Proposed	90.798	89.4118	81.640	85.346	198.45	6.35
Bi-flow	K-NN	96.976	88.321	82.333	85.221	54.80	11.90
	NB	91.188	91.996	79.375	85.220	6.64	3.82
	DT	97.039	95.479	80.998	87.644	6.10	2.89
	RF	97.979	96.391	84.373	89.982	8.39	3.97
	Proposed	98.127	95.109	86.717	90.718	163.27	4.44
Uni-flow	K-NN	96.992	96.973	76.111	85.284	46.88	10.98
	NB	70.765	69.185	70.988	70.075	6.49	3.46
	DT	96.171	96.140	75.899	84.828	7.70	2.98
	RF	95.297	95.117	73.995	83.236	8.29	3.37
	Proposed	97.084	94.761	86.435	90.605	143.38	4.58

**Table 19:** Comparison with other deep learning models.

Models	Accuracy	F1 Score
LSTM [24]	0.9337	0.9328
GRUs [24]	0.9608	0.9577
Proposed	0.9713	0.9599

## Data Availability

The publicly available data set can be found at: https://joseaveleira.es/dataset and https://ieee-dataport.org/open-access/mqtt-iot-ids2020-mqtt-internet-things-intrusion-detection-dataset.
